# In vivo tissue clearing with tartrazine and other dye molecules

**DOI:** 10.1038/s42003-026-10610-4

**Published:** 2026-07-03

**Authors:** Victoria Crunkleton, Guosong Hong

**Affiliations:** 1 Department of Materials Science and Engineering, Stanford University, Stanford, CA USA; 2 Wu Tsai Neurosciences Institute, Stanford University, Stanford, CA USA

**Keywords:** Biological fluorescence, Optical imaging

## Abstract

Tartrazine, along with many other absorbing molecules, has recently emerged as a promising tissue-clearing agent for achieving transient and reversible optical transparency in live animals. In this mini-review, we describe the fundamental physical origins of tissue opacity, extract engineering principles guiding the design of next-generation dye-based clearing agents, and summarize recent advances in dye-enabled in vivo tissue clearing. We also discuss current challenges and offer forward-looking perspectives to inform future research.

## Abstract (web summary)

A review summarizes the emerging field of dye-enabled in vivo optical tissue clearing, encompassing its fundamental physical mechanisms, engineering design principles, and diverse biological and biomedical applications developed worldwide.

## Introduction

Light is used in a wide range of methods in biology and medicine, such as fluorescence imaging, optogenetics, and photopharmacology^1–5^. A critical challenge for all light-based methods in living systems faces a fundamental limitation: biological tissues are inherently opaque^6^. As light travels through tissue, it is strongly scattered and absorbed, preventing photons from penetrating deeply into the body^7^. This limitation frequently necessitates invasive procedures, such as surgically exposing tissues^8^, implanting optical windows^9^, or inserting optical fibers and endoscopes^10–12^. Albeit effective, these approaches come at the cost of invasiveness, discomfort, and limited applicability, highlighting a central challenge in applying and translating optical technologies to living systems.

Biological tissues are opaque due to both scattering and absorption of light, with scattering typically an order of magnitude stronger and thus the primary barrier to deep optical access in the visible spectrum^13^. Light scattering arises from differences in refractive indices (RI) between low-RI aqueous components (e.g., intracellular and extracellular fluids) and high-RI lipid and protein components (e.g., plasma/organelle membranes and collagen fibers in the extracellular matrix)^6^. Therefore, conventional “tissue-clearing” methods either remove water to “match up” the RI (e.g., hydrophobic tissue clearing) or remove lipids to “match down” the RI (e.g., hydrogel-based tissue clearing)^14–17^. However, the removal of tissue components essential for life and the use of toxic substances make these methods fundamentally incompatible with living systems^18^.

**Fig. 1:**
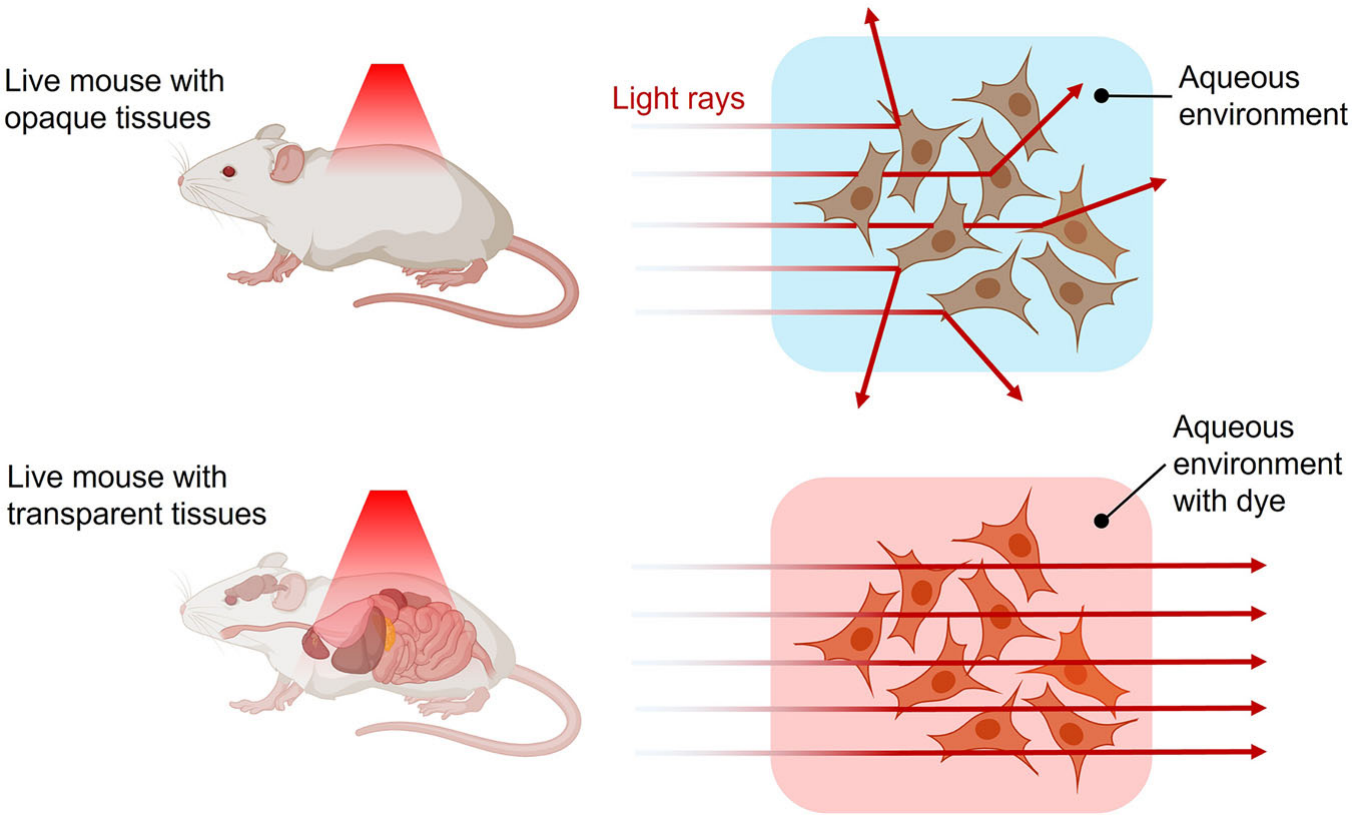
Overview of in vivo tissue clearing with absorbing dye molecules to achieve optical transparency in live animals. Created in BioRender. Hong, G. (2026) https://BioRender.com/qmgu2tx.

Recently, our lab demonstrated the world’s first live transparent mouse using strongly absorbing molecules^19–21^. We leveraged the Kramers–Kronig relations to achieve RI matching across tissue components by tuning their absorption properties with dye molecules (Fig. 1)^22,23^. This approach enabled visualization of deep-seated biological structures and dynamic processes in living mice. Since its publication, this work has been widely reproduced and extended by laboratories around the world^24–46^. These studies include successful demonstrations in which tartrazine is applied to render skin transparent for optical coherence tomography (OCT) and photoacoustic microscopy^24,27–31,34^, as well as to make the skull transparent for laser speckle contrast imaging^25^.

Importantly, the same underlying physical principles can be generalized beyond tartrazine, enabling other strongly absorbing molecules—such as fluorescein^47,48^, indocyanine green^49^, and ampyrone^24,50^—to be repurposed as in vivo tissue-clearing agents. Besides these exogenous absorbing molecules, endogenous chromophores, such as hemoglobin, have also served as intrinsically biocompatible clearing agents^51,52^. For example, hemolysis expels hemoglobin from erythrocytes into blood plasma, providing RI matching between the two to reduce scattering in blood^53–55^. Additionally, tissue has been cleared using hemoglobin solutions to achieve RI matching between collagen and aqueous components^56^. Although hemoglobin absorbs in the visible and thus limits clearing effect to the near-infrared spectrum, it still enhances light transmission in tissue, serving as a conceptual bridge toward endogenous clearing strategies.

### Physical insights: why do different tissue components have different refractive indices?

Any meaningful attempt to improve how light penetrates living tissue must begin with a fundamental understanding of *why* biological tissues are opaque in the first place. A seminal review by Tuchin showed that biological tissues are intrinsically heterogeneous in *both chemical composition and optical properties*, and that tissue heterogeneity underlies light scattering, preventing ballistic photon propagation (Fig. 1)^6^. Specifically, the RI values of biological tissue components are bounded at the low end by water, which has an average RI of approximately 1.33 in the visible spectrum, and at the high end by mineralized substances such as hydroxyapatite, whose RI ranges from about 1.60 to 1.70 in the visible. Aqueous components of the body, includingcytoplasm and extracellular fluids, have RI values close to that of water (1.35–1.36). In contrast, non-aqueous components, such as cell membranes, nuclei, and other lipid- and protein-rich structures, exhibit substantially higher RI values, typically in the range of 1.40–1.50. Light scattering arises from this RI heterogeneity at length scales comparable to the wavelength of light (Mie scattering) as well as at moderately subwavelength scales (Rayleigh scattering)^57^. In other words, when heterogeneous structures spanning length scales from nanometers (such as the thickness of plasma and organelle membranes) to hundreds of nanometers (including endosomes, mitochondria, and chromatin domains) are embedded in an aqueous background, light scattering arises at the interfaces between these refractive-index mismatched components.

A deeper and more fundamental question is why different tissue components possess distinct RIs in the first place. Put simply, why does water have a much lower RI than lipids, proteins, and nucleic acids in the visible spectrum? Addressing this question requires viewing the RI as a wavelength-dependent, complex quantity, which leads to several important insights rooted in the fundamental physics of light-matter interactions and the mathematics of complex analysis.

First, the RI consists of both a real and an imaginary component. The real component, $n^{\prime} (\lambda )$, corresponds to the RI commonly discussed in the tissue-clearing literature, while the imaginary component, $n^{\prime\prime}(\lambda )$, describes optical absorption. The complex RI, $N(\lambda )$, can be expressed as follows:

1 $$N(\lambda )=n^{\prime} (\lambda )+{in}^{\prime\prime}(\lambda )$$

where $i$ is the imaginary unit, and the sign of the imaginary component follows the convention ${e}^{-i\omega t}$ for time-harmonic fields.

**Fig. 2:**
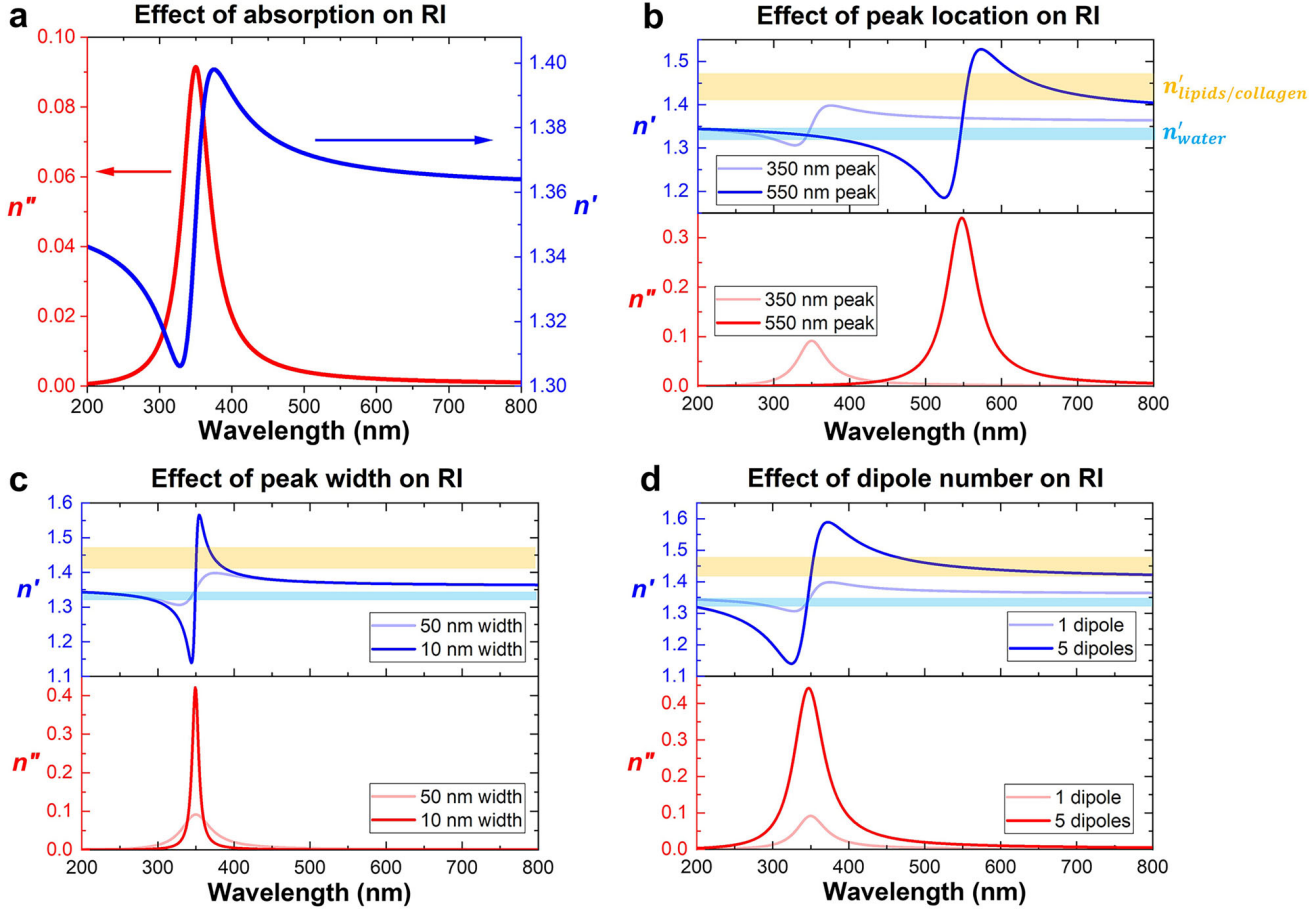
Strategies for tuning the refractive index of water. **a** Simulation of the real (*n*’) and imaginary (*n*′′) components of the complex refractive index resulting from dissolving a hypothetical absorbing molecule in water, illustrating how absorption tunes the refractive index. Parameters for simulating the absorbing molecule as a Lorentzian oscillator: peak location—350 nm; peak width—50 nm; number of dipoles per molecule—1; concentration—0.5 M. **b** Simulation results illustrating the effect of shifting the absorption peak from 350 to 550 nm while keeping all other parameters in the Lorentz oscillator model constant. **c** Simulation results illustrating the effect of tuning the peak width from 50 to 10 nm while keeping all other parameters in the Lorentz oscillator model constant. **d** Simulation results illustrating the effect of tuning the number of light-induced oscillating dipoles per molecule from 1 to 5 while keeping all other parameters in the Lorentz oscillator model constant. The yellow and cyan shaded regions in (**b–d**) indicate the refractive-index ranges of lipids/proteins (the target range for modulating the refractive index of water) and pure water in the visible spectrum, respectively. MATLAB code for simulation is available in the Supplementary Code.

Second, both components depend on wavelength. In spectral regions where absorption is negligible, the real component exhibits a characteristic normal dispersion behavior, varying smoothly with wavelength (Fig. 2a).

Lastly, and most importantly in the context of dye-based tissue clearing, the real and imaginary components of the complex refractive index are causally linked through the Kramers–Kronig relations, as described below:

2 $$n^{\prime} (\lambda )=1+\frac{2}{\pi }P.V.{\int }_{0}^{\infty }n^{\prime\prime}(\lambda^{\prime} ){\left[\lambda^{\prime} (1-\frac{\lambda ^{{\prime} ^{2}}}{{\lambda }^{2}})\right]}^{-1}d\lambda ^{\prime}$$

where P.V. is the Cauchy principal value of the integral^23^. Intuitively, Eq. (2) reveals that absorption at one wavelength must influence refraction at other wavelengths because a material’s optical response cannot occur before the light interacts with it, and this requirement of causality mathematically links energy loss (absorption) to phase delay (refractive index) through the Kramers–Kronig relations.

Applying the Kramers–Kronig relations between absorption and refraction in Eq. (2), the real part of the RI at a given visible wavelength is positively and causally determined by an integral over the material’s absorption spectrum at shorter wavelengths, especially absorption in the ultraviolet (UV) region, where the kernel is positive. Therefore, one can conclude that different tissue components exhibit distinct RI (more precisely, different values of $n^{\prime}$) in the visible spectrum because they possess different absorption spectra in the ultraviolet (UV) region. In particular, water must have a lower spectrally integrated UV absorption than lipids and proteins in order to give rise to its lower RI in the visible. The absorption of light by a specific molecule can be understood using the Lorentz oscillator model, which describes absorption strength in analogy to the oscillation amplitude of a driven spring:

3 $${\varepsilon }_{r}={N}^{2}={\varepsilon }_{\infty }+\frac{{\omega }_{p,\,{lowest}}^{2}}{{\omega }_{0,\,{lowest}}^{2}-{\omega }^{2}-i{\gamma }_{{lowest}}\omega }$$

where ${\varepsilon }_{r}$ is the relative permittivity of a given molecule, which is equal to the square of its complex RI, $N$. In addition, ${\varepsilon }_{\infty }$ is the high-frequency dielectric constant that contains the contributions of all higher-frequency oscillators^57^. Furthermore, ${\omega }_{p,{lowest}}$ and ${\omega }_{0,{lowest}}$ are the plasma frequency and resonance frequency, respectively, of the equivalent oscillator with the lowest resonant frequency, corresponding to the longest-wavelength absorption peak of the molecule of interest, just before the visible spectrum. Moreover, ${\gamma }_{{lowest}}$ is the damping constant of this lowest-resonant-frequency oscillator, and $\omega$ is the operating frequency, related to the freespace wavelength of incident light, $\lambda$, as follows:

4 $$\omega =\frac{2\pi c}{\lambda }$$

where $c$ is the speed of light in vacuum.

The interaction of a given molecule with light at wavelengths just to the right (red-shifted) of the longest-wavelength absorption peak can be described by the following expression for $n^{\prime}$:

5 $$n^{\prime} \approx \sqrt{{\varepsilon }_{\infty }}+\frac{1}{\sqrt{{\varepsilon }_{\infty }}}\bullet \frac{{\omega }_{p,\,{lowest}}^{2}\varDelta \omega }{{\omega }_{0,{lowest}}\left(4{\left(\varDelta \omega \right)}^{2}+{\gamma }_{{lowest}}^{2}\right)}$$

where $\varDelta \omega ={\omega }_{0,{lowest}}-\omega$, representing the amount of red shift of the incident light frequency relative to the longest-wavelength absorption peak prior to the visible spectrum^19^.

Equation (5) yields an important takeaway: if one neglects the contribution of absorption at extremely high frequencies to $n^{\prime}$—a reasonable approximation given the inability of electrons to follow very rapidly oscillating electric fields—the real refractive index in the visible spectrum is primarily determined by the position of the last major UV absorption peak preceding the visible range, namely ${\omega }_{0,{lowest}}$. Water molecules are dominated by absorption from the O–H σ bond at wavelengths below ~100 nm in the extreme ultraviolet (EUV)^58^, whereas lipids exhibit absorption bands extending to ~200 nm due to carbonyl groups^59^, proteins absorb around 280 nm owing to the aromatic side chains of amino acids such as tryptophan and tyrosine^60^, and nucleic acids absorb near 260 nm due to purine and pyrimidine bases^61^. According to Eq. (5), it is not surprising that water has the lowest RI in the visible spectrum due to the highest ${\omega }_{0,{lowest}}$, i.e., shortest absorption wavelengths in the EUV. In contrast, tissue components rich in lipids, proteins, and nucleic acids—such as plasma membranes, collagen fibers, and cell nuclei—exhibit substantially higher RIs, due to their lower ${\omega }_{0,{lowest}}$, i.e., longer absorption wavelengths in the near ultraviolet (NUV, 180–400 nm).

In summary, the magnitude of a material’s RI in the visible spectrum is causally determined by its absorption at shorter wavelengths, particularly in the ultraviolet (UV), in accordance with the Kramers–Kronig relations^23^. As a result, water exhibits a lower visible RI than most biomolecules precisely because it absorbs much less strongly in the UV. This insight may guide the design and realization of genetically engineered transparency, in which tissues are programmed to produce biomolecules with tailored ultraviolet absorption profiles and appropriately balanced integrated UV absorption, thereby leveling the RIs of key tissue components in the visible spectrum.

### Engineering insights: is refractive index modulation in biological tissues possible?

This physical principle revealed above, in turn, suggests an engineering strategy: the RI of water can be increased by deliberately introducing UV-absorbing molecules, thereby compensating for water’s intrinsic lack of absorption and bringing its visible RI closer to that of lipids and proteins. The RI change from absorbing molecules can be approximated by the linear relation, $\triangle n^{\prime} =\beta \cdot c$, where $\beta$ is defined as the change in $n^{\prime}$ of the medium (e.g., water) per molar concentration, *c*, of the dissolved clearing agent. Using this definition, Eq. (5) can be further modified to yield the expression for $\beta$:

6 $$\beta =\frac{\triangle n^{\prime} }{c}=\frac{{{ZN}}_{A}{e}^{2}\varDelta \omega }{{n}_{0}^{\prime} m{\varepsilon }_{0}{\omega }_{0,{lowest}}\left(4{\left(\varDelta \omega \right)}^{2}+{\gamma }_{{lowest}}^{2}\right)}$$

where $Z$ is the effective number of electrons contributing to the Lorentz oscillators per molecule of the clearing agent, *N*_A_ is Avogadro’s constant, *e* and *m* are the charge and mass per electron, ${n}_{0}^{\prime}$ is the real refractive index of pure medium, and ${\varepsilon }_{0}$ is vacuum permittivity.

Equation (6) summarizes key principles of designing and developing more efficient agents for tissue clearing:

First, given the ${\omega }_{0,{lowest}}^{-1}$ scaling, molecules with lower resonance frequencies (that is, longer absorption wavelengths) are more effective at increasing the RI of the medium (Fig. 2b). At the same time, these molecules should have negligible absorption at the operating wavelength to avoid attenuation of photons used for imaging or light delivery.

Second, given the ${\gamma }_{{lowest}}^{-2}$ scaling, molecules with smaller damping constants—and therefore sharper absorption peaks—are more effective at increasing the RI of the medium (Fig. 2c). It should be noted that this principle applies only in the near-resonance regime, namely when the operating wavelength used for imaging or light delivery lies sufficiently close to the absorption peak wavelength.

Third, given the *Z* scaling, absorbing molecules with multiple light-induced oscillating dipoles are more effective at increasing the RI of the medium (Fig. 2d). Because Lorentzian oscillator molecules in the visible spectrum are typically π-conjugated systems, this scaling implies that molecules composed of multiple, distinct conjugated units can serve as more efficient tissue-clearing agents.

Equation (6) should be viewed not only as a guiding principle for designing and developing absorbing dye molecules in tissue clearing, but also as a unifying framework for understanding the effectiveness of many conventional clearing agents identified over the past three decades, including glycerol, sucrose, sorbitol, and antipyrine^62–65^. These conventional clearing agents have been used for in vivo optical clearing through multiple mechanisms, including refractive index matching, dehydration, and collagen reorganization within the extracellular matrix^17^. Notably, these conventional clearing agents can also be regarded as “dye-like” molecules in the sense that they exhibit absorption in the deep UV spectrum. Because their absorption occurs at extremely short wavelengths—likely intended to be completely transparent in the visible spectrum—they are relatively inefficient at increasing the RI of the aqueous medium. As a result, conventional clearing agents must be used at very high concentrations, often approaching complete replacement of water, to achieve sufficient RI matching. This requirement leads to dehydration and removal of essential tissue components, making such approaches incompatible with living systems. Nonetheless, these deep-UV absorbing molecules remain highly effective for RI matching in UV imaging and treatments, particularly when the operating wavelength is close to the absorption resonance^66–68^.

In contrast, dye molecules with strong absorption in the NUV or visible spectrum can substantially raise the RI of water without displacing the majority of water molecules, thereby preserving the native aqueous environment and inducing minimal perturbation to biological function (Supplementary Table 1). As an illustrative example, tartrazine can raise the RI of water by a $\varDelta n^{\prime}$ of 0.12 at a concentration of 0.62 M, corresponding to a mass fraction of ~30 wt% (with ~70 wt% remaining water)^19,20^. Remarkably, recent studies have shown that even isotonic tartrazine solutions (75 mM, ~4 wt%) can produce significant tissue-clearing effects^34^. These studies reveal a $\beta$ value of 0.19 M^−1^ for tartrazine at ~500 nm based on the definition of $\beta$ in Eq. (6). Moreover, Eq. (6) predicts that dye molecules with absorption peaks at even longer wavelengths—such as indocyanine green (ICG)—should enable in vivo tissue clearing at still lower concentrations. This prediction has been experimentally validated, with effective clearing achieved using ICG at concentrations as low as 24 mM (~2 wt%)^49^. Specifically, the intense absorption peak of ICG at ~780 nm results in a higher $\beta$ value of 0.56 M^−1^ at 830 nm^49^. Based on the design principles illustrated in Fig. 2, more efficient tissue-clearing agents are expected to emerge, particularly those featuring sharper absorption linewidths, multiple resonant dipoles per molecule, and absorption peaks shifted toward longer wavelengths while remaining outside the imaging window.

While Eq. (6) suggests clear design strategies for enhancing RI modulation, it is important to recognize both fundamental and practical limits. From a physical standpoint, the absorption strength of a molecule is constrained by its oscillator strength, which is ultimately limited by the number of electrons available for light-matter interaction at a given frequency. Specifically, the absorption cross section of an absorbing molecule is fundamentally upper-bounded on the order of ~${\lambda }^{2}$^69^, which in turn sets a theoretical lower bound on the concentration required to achieve a given level of RI modulation. In practice, however, this theoretical limit is relatively permissive. More restrictive constraints arise from factors such as solubility, biocompatibility, and delivery efficiency, which limit the maximum usable dye concentration and therefore the achievable RI change. For near-resonance clearing, it is necessary to consider competition with absorption loss. For each operating wavelength, an optimal dye concentration can, in principle, be determined by identifying the local minimum of the sum of the scattering and absorption coefficients for a given tissue of interest, both of which depend on the concentration and absorption profile of the clearing dye. Taken together, these considerations indicate that, while substantial RI modulation is attainable, careful molecular design and screening are required to approach the limits imposed by both fundamental physics and practical implementation.

### Biological insights: what does in vivo tissue clearing enable?

**Fig. 3:**
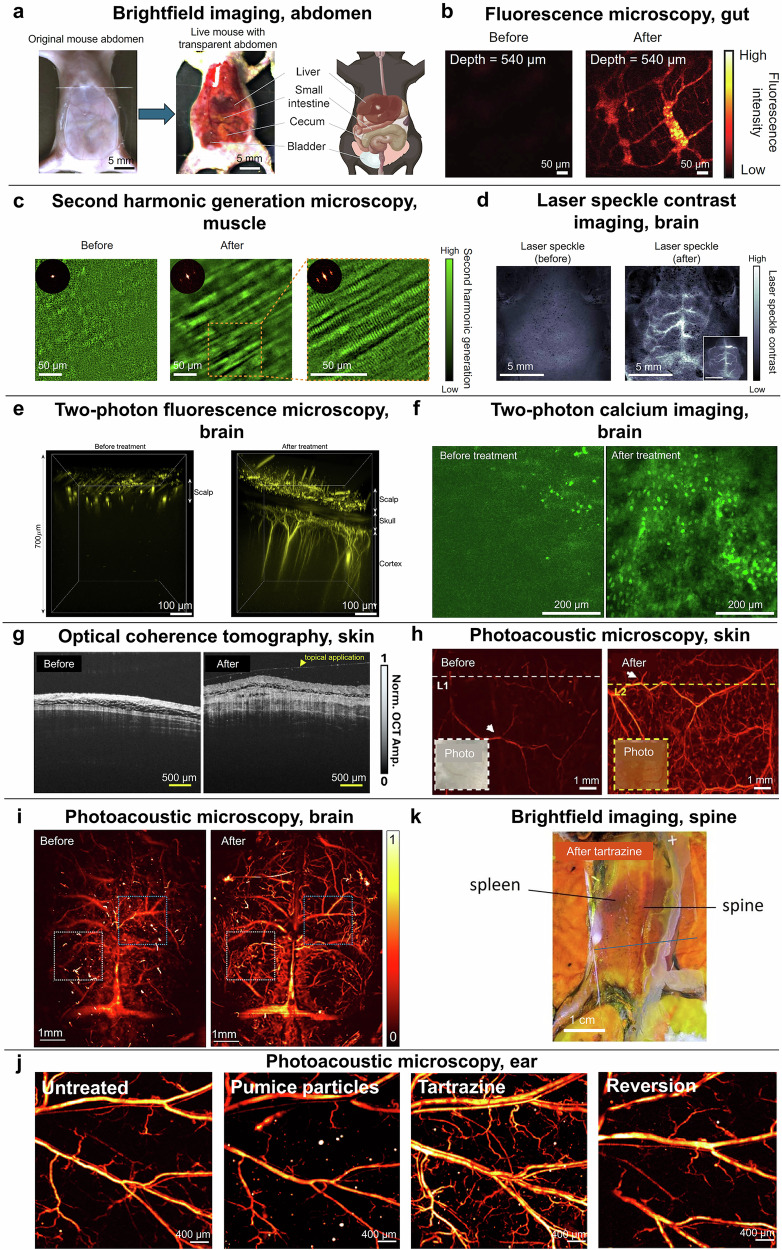
Representative examples of dye-enabled in vivo tissue clearing. **a** Visualizing internal organs through a transparent abdomen. **b** Microscopic imaging of enteric neurons through a transparent abdomen. **c** Microscopic imaging of muscle sarcomeres through cleared skin. **d** Visualizing cerebral vasculature in the brain through cleared scalp and intact skull. **e** Microscopic imaging of cortical neurons in the brain through cleared scalp and intact skull. **f** Microscopic imaging of the calcium signal from cortical neurons in the brain through the cleared scalp and intact skull. **g** OCT imaging of skin-layer architecture in the abdomen after clearing the abdominal skin. **h** Photoacoustic microscopy of abdominal vasculature after clearing the skin. **i** Photoacoustic microscopy of cerebrovasculature through the cleared scalp and skull. **j** Photoacoustic microscopy of ear microvasculature after clearing the skin. **k** Visualizing spine and spleen through a transparent dorsal skin. **a**–**d** are reproduced from ref. ^19^ with permissions from publishers. **e**, **f** are reproduced from ref. ^50^ with permissions from publishers. **g**, **h** are reproduced from ref. ^24^ with permissions from publishers. **i** is reproduced from ref. ^27^ with permissions from publishers. **j** is reproduced from ref. ^29^ with permissions from publishers. **k** is reproduced from refs. ^26,38^ with permissions from publishers.

The dye-based in vivo clearing method enables a transient and reversible transparency window within the living body, without significant dehydration or irreversible removal of essential tissue components. Since its introduction, this approach has been applied to a wide range of imaging modalities and biological contexts (Box 1 and Supplementary Table 1). Some of the representative examples include noninvasive, naked-eye visualization of internal organs such as the liver and intestines using brightfield imaging (Fig. 3a)^19,20^; dynamic microscopic imaging of enteric neurons during gut motility through a cleared abdominal wall using widefield fluorescence microscopy (Fig. 3b)^19,20^; static microscopic imaging of muscle sarcomeres through intact skin in the mouse hindlimb using second-harmonic generation microscopy (Fig. 3c)^19,20^; laser speckle contrast imaging of cerebral hemodynamics through the intact scalp and skull (Fig. 3d)^19,20^; chronic imaging of YFP-labeled neurons through the cleared scalp and skull in the developing mouse cortex (Fig. 3e)^50^; dynamic imaging of GCaMP-labeled neurons through cleared scalp and skull in awake mice (Fig. 3f)^50^; optical coherence tomography of live mouse abdominal tissue (Fig. 3g)^24^; photoacoustic microscopy of live mouse abdominal tissue (Fig. 3h)^24^; photoacoustic microscopy of the live mouse brain through cleared scalp and skull (Fig. 3i)^27^; photoacoustic imaging of live mouse ear vasculature (Fig. 3j)^29^; and noninvasive, naked-eye visualization of the spine and spleen through intact dorsal skin in live mice (Fig. 3k)^26,38^.

Dye-enabled in vivo tissue clearing has enabled important biomedical insights that are otherwise difficult or impossible to obtain. From a biological perspective, this approach enables, for the first time, visualization of enteric neuronal activity during unobstructed gut motility, in contrast to conventional abdominal windows that disrupt normal gut motion and limit studies of gut physiology and the enteric nervous system in their native state^19,20^. Another insight in developmental biology has been obtained through noninvasive in vivo imaging of embryonic development by clearing the pregnant dam’s skin with tartrazine, enabling longitudinal in utero imaging without conventional invasive interventions^70^. From a diagnostic standpoint, in vivo clearing combined with OCT allows biopsy-free visualization of striated skin structures with resolution comparable to H&E histology, paving the way for noninvasive skin diagnostics^24,71^. From a therapeutic perspective, in vivo clearing enhances light penetration, improving the efficacy of light-based treatments such as photodynamic therapy and photochemical tissue bonding^39,72^.

Dye molecules with appropriate absorption properties can also be employed for ex vivo tissue clearing, offering an alternative to conventional clearing agents that are often used at concentrations too high to preserve tissue integrity and size. For example, tartrazine has been used to enable transscleral OCT imaging in porcine eyes with thick sclera^31^. In addition, tartrazine exhibits unique advantages for enhancing deep-tissue Raman biomolecular sensing, owing to both its RI matching capability and its ability to suppress tissue autofluorescence^32^. Finally, tartrazine has recently been demonstrated to function effectively as a clearing agent for fluorescence lifetime imaging, benefiting from both its RI matching and its intrinsically ultrashort fluorescence lifetime^33^.

Box 1 Key developments and open challenges Key developments

1. Strongly absorbing dye molecules can function as effective clearing agents to achieve in vivo optical transparency. This strategy is general and applicable to a wide range of dyes, including—but not limited to—tartrazine, fluorescein, indocyanine green, and antipyrine.
2. Dye-enabled in vivo tissue clearing has been used for macroscopic, naked-eye visualization of internal organs—including the liver, intestines, bladder, spleen, and spine—through the skin and other overlying tissues in live mice.
3. Dye-enabled in vivo tissue clearing has been utilized for microscopic imaging of deep-seated cellular structures and dynamic biological activities in live mice, including cortical neurons in the brain, enteric neurons in the gut, muscle sarcomeres in the hindlimb, and skin-layer architecture in the abdominal region.
4. Dye-enabled in vivo tissue clearing has been utilized for mesoscopic imaging of deep-seated vasculature and hemodynamics in live mice, including laser speckle contrast imaging of cerebral blood flow through the intact scalp and skull, as well as photoacoustic imaging of ear vasculature.
5. Dye-enabled tissue clearing has also been applied to transscleral imaging of the eye, deep-tissue Raman sensing, and fluorescence lifetime imaging.

Open challenges

1. Despite being more efficient than conventional tissue-clearing agents, existing absorbing dye molecules still must reach relatively high concentrations to effectively match the RI of lipid-rich tissue components. This challenge may be mitigated through the use of transient and reversible clearing strategies, recent demonstrations of substantial clearing using isotonic dye solutions, and rational design of more efficient absorbing dye molecules with potential membrane permeability.
2. Topically applied dye molecules exhibit limited diffusion into deep tissues, thereby constraining the achievable depth of optical clearing. Combining topical delivery with physical and chemical percutaneous enhancement strategies, as well as exploring alternative routes of administration, offers promising pathways to enable deeper in vivo tissue clearing.
3. Due to heterogeneity in RI across different tissue components, using a single dye concentration with a fixed RI cannot completely eliminate scattering, but can only minimize it. Engineering cells to produce dye molecules in different compartments, with RI tailored to the local environment, represents a promising approach to achieving complete transparency.
4. The free diffusion and continuous metabolic clearance of delivered dye molecules cause optically cleared windows to gradually diminish over time. In contrast, covalent crosslinking of dye molecules to appropriate tissue components, as well as endogenous synthesis of absorbing molecules, represent promising strategies for achieving permanent or long-lasting optical transparency.

### Outlook and perspectives

Despite these exciting developments, important challenges remain. Although dye-based clearing agents are substantially more effective than conventional agents in terms of molar concentration and mass fraction, relatively high concentrations are still required to achieve complete RI matching. For example, tartrazine must reach ~0.6 M in water (~30 wt%) to match the RI of lipid-rich tissue components. While this is already much lower than concentrations used in traditional clearing agents, it still raises biosafety concerns, including hypertonic stress, irritation, and inflammation. To assess this, we recently showed that cultured human embryonic kidney (HEK) cells maintain >95% viability after 45 min of continuous exposure to hyperosmotic tartrazine^73^. This suggests that short-term exposure, the biocompatibility of tartrazine, and its potential membrane permeability help mitigate adverse effects at effective concentrations. These findings support the use of transient tartrazine exposure for in vivo tissue clearing: when dyes are topically administered transiently and subsequently washed out to reverse the clearing effect, no significant cellular or tissue damage has been observed in live mice^19,20,46,50^. An exciting recent study has shown that isotonic tartrazine solutions at much lower concentrations (75 mM, ~4 wt%) can still produce substantial enhancements in imaging penetration depth, offering a promising path toward effective clearing at reduced dosages^34^. However, a comprehensive evaluation of the biological effects of tartrazine and other dye molecules will require further, more detailed studies. We anticipate that continued development of more efficient absorbing molecules—guided by the physical design principles rooted in the Kramers–Kronig relations (Fig. 2)—will yield next-generation clearing agents that operate at millimolar or even lower concentrations. Such advances would further improve biocompatibility and open the door to long-term in vivo tissue clearing applications.

Another challenge lies in the delivery efficiency of dye-based clearing agents for in vivo tissue clearing. To date, almost all reported studies apply tartrazine and other absorbing dye molecules topically to the skin, which inherently limits the achievable depth of tissue clearing due to the restricted diffusion distance of these molecules. Notably, among reported dye-based in vivo clearing agents, tartrazine, fluorescein, and indocyanine green are all anionic molecules, which are generally challenging to deliver transdermally^19,47,49^. Neutral dyes, such as ampyrone, exhibit improved skin penetration^38,50^; however, they are less widely used because most neutral dyes have limited water solubility, which restricts their ability to reach concentrations required for effective RI modulation. Cationic dyes can, in principle, electrostatically interact with negatively charged membranes, potentially facilitating transdermal delivery^74^; however, their nonuniform distribution across cellular compartments and specific binding to membranes and proteins may increase RI heterogeneity rather than promote homogenization. Beyond charge, molecular weight represents an additional factor that may constrain diffusion and transdermal delivery. To address these challenges, percutaneous enhancement strategies—such as physical skin abrasion^24,29^ or the use of depilatory agents (e.g., Nair) to disrupt disulfide bonds in the keratinized epidermal layer^19,20^—can facilitate more efficient penetration of these dye molecules through the skin. Future advances may include the development of more effective physical and chemical percutaneous enhancers, such as transdermal microneedle arrays^75^; ultrasound patches for enhancing transdermal transport^76^; rational design of new absorbing molecules with balanced water-oil amphiphilicity; and the use of chemical penetration enhancers with FDA-approved safety profiles^77^. Lastly, direct delivery of dye molecules into local tissue via syringe injection or microfluidic channels may provide viable alternatives to topical application for deep-tissue optical imaging.

The physical principles underlying dye-enabled tissue clearing are so general that many additional dye molecules are expected to be leveraged as clearing agents in the coming years. Indeed, it has already been demonstrated that fluorescein—a widely accessible contrast agent with a long history of clinical use—can be repurposed as a reversible clearing agent for transscleral OCT imaging of porcine and human donor eyes^47,48^. In addition, another FDA-approved near-infrared (NIR) contrast agent, ICG, has recently been shown to function as an effective clearing agent for in vivo murine skin^49^. Given that numerous absorbing dyes beyond tartrazine are already commonplace in clinical practice, and that many fluorophores developed over the past decades for fluorescence imaging are themselves chromophores with strong absorption properties, it is reasonable to envision a future in which many of these molecules are repurposed as tissue-clearing agents. In such a framework, each dye would possess an optimal transparency window determined by its near-resonant dispersion behavior, as illustrated in Fig. 2. In particular, for fluorescence imaging, one can envision a strategy in which each fluorophore is rationally paired with a complementary dye molecule—potentially another well-established fluorophore—whose absorption spectrum is slightly blue-shifted relative to the excitation wavelength of the imaging fluorophore. In such a paired system, the complementary dye would experience minimal excitation and fluorescence due to off-resonance while providing effective RI modulation, thereby acting as a clearing agent that enables deeper and less invasive fluorescence imaging in living tissues. Of note, the complementary dye must exhibit minimal absorption at the excitation wavelength of the fluorophore, as even slight absorption could induce undesirable photothermal and phototoxic effects. Nonetheless, in certain cases, suppressing multiple-scattered photons can enhance imaging contrast, even at the expense of absolute signal intensity^78,79^.

Lastly, due to free diffusion and systemic clearance of delivered dye molecules, any optically cleared window is inherently transient unless dyes are continuously supplied. While such reversible and re-establishable clearing windows are often sufficient for accessing otherwise inaccessible information from deep tissues in chronic studies, longer-lasting or permanent transparency may be desirable for certain applications. In addition, systemic distribution of locally applied clearing agents may lead to off-target effects, increasing the pharmacological burden on other tissues and organs. One potential strategy for sustained clearing involves covalently crosslinking absorbing molecules to extracellular matrix components, thereby immobilizing the clearing agents and maintaining long-term RI matching. In addition, insights from the biology of inherently transparent species, such as zebrafish larvae and glass frogs^80–82^, suggest alternative approaches. Inspired by these systems, it is now conceivable to genetically encode and express absorbing molecules—such as chromoproteins or small-molecule biopigments—within mammalian cells to actively modulate their RI profiles. Finally, the fundamental physical framework provided by the Kramers–Kronig relations points to an exciting future in which genetically encoded transparent mammals could serve as transformative model systems for studying biological processes and developing new therapies in living subjects across the full three-dimensional body.

## Supplementary information

Supplemental Information
