# Supplementary material for: In vivo tissue clearing with tartrazine and other dye molecules
Source: Commun Biol. 2026 Jul 3;9:895. doi: 10.1038/s42003-026-10610-4 (PMC13332022; doi:10.1038/s42003-026-10610-4)
Supplement: Supplementary file 1 — Supplemental Information [file 42003_2026_10610_MOESM1_ESM.pdf]

Supplementary Materials for

In vivo tissue clearing with tartrazine and other dye molecules

Victoria Crunkleton[1,2] and Guosong Hong[1,2*]

[1]Department of Materials Science and Engineering, Stanford University, Stanford, CA, USA

[2]Wu Tsai Neurosciences Institute, Stanford University, Stanford, CA, USA

*To whom correspondence may be addressed. Email: guosongh@stanford.edu

Table of Contents:

Supplementary Table……………………………………………………………………………………2

Supplementary Code…………………………………………………………………….……………...3

Supplementary References…………………………………………………………………………….5

Supplementary Table

Supplementary Table 1. Properties and applications of dye molecules utilized as in vivo optical clearing agents.

Dye molecules as clearing agents	Chemical structure	λ_{max} (nm)*	Applicable transparency ranges	Experimentally demonstrated applications	Refs
**Tartrazine**	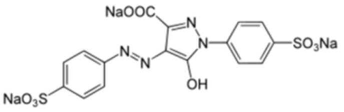	428	red & near-infrared	Brightfield imaging, widefield fluorescence microscopy, second harmonic generation microscopy, laser speckle contrast imaging, two-photon fluorescence microscopy, optical coherence tomography, optical coherence angiography, photoacoustic microscopy, photodynamic therapy, photochemical tissue bonding	1–23
**Indocyanine green**	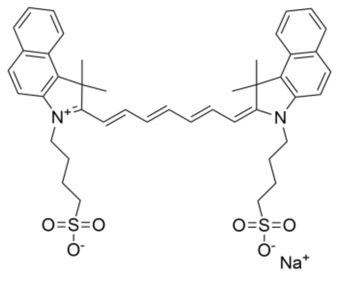	780	near-infrared	Optical coherence tomography	24
**Fluorescein**	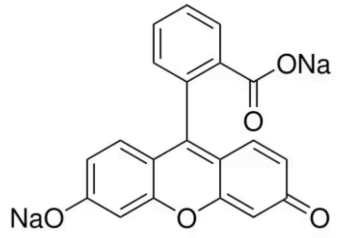	490	red & near-infrared	Optical coherence tomography	25,26
**Ampyrone**	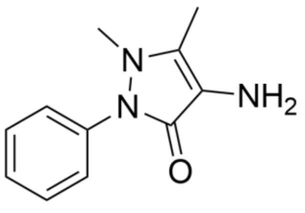	290	visible	Brightfield imaging, two-photon fluorescence microscopy, two-photon calcium imaging, optical coherence tomography, photoacoustic microscopy	1,11,15,27

* The peak absorption is shown for that with the longest wavelength, corresponding to the Lorentzian oscillator with the lowest resonant frequency, which has a stronger effect on the refractive index at the imaging wavelengths, according to Eqs. (3), (5), and (6) in the main text.

Supplementary Code

```
% All rights reserved by the Hong Lab at Stanford.
clear; close all; clc;
% ----------------- Constants -----------------
c    = 2.99792458e8;          % m/s
eps0 = 8.8541878188e-12;      % F/m
e    = 1.602176634e-19;       % C
me   = 9.1093837139e-31;      % kg
NA   = 6.02214076e23;         % 1/mol
% ----------------- Model parameters -----------------
number_dipole_per_molecule = 1;     % effective oscillator-strength
scaling per molecule
concentration_M = 0.5;              % mol/L
% Convert concentration to number density (1/m^3)
N = concentration_M * 1000 * NA;    % (mol/L)*(1000 L/m^3)*(molecules/mol)
% Effective oscillator strength parameter (Lorentz oscillator amplitude)
omega_p2 = N * number_dipole_per_molecule * e^2 / (me * eps0);   %
(rad/s)^2
peak_wavelength_nm = 550;           % nm (absorption peak location)
lambda0 = peak_wavelength_nm * 1e-9;
omega0  = 2*pi*c/lambda0;           % rad/s
% Define linewidth in an explicit way (approx. mapping from wavelength
width to gamma)
peak_width_nm = 50;                 % nm (user-defined; interpreted as
approximate FWHM in wavelength)
lambda_plus  = (peak_wavelength_nm + peak_width_nm/2) * 1e-9;
lambda_minus = (peak_wavelength_nm - peak_width_nm/2) * 1e-9;
omega_plus  = 2*pi*c/lambda_plus;   % rad/s
omega_minus = 2*pi*c/lambda_minus;  % rad/s
% Approximate gamma as FWHM in angular frequency
gamma = abs(omega_minus - omega_plus);  % rad/s
% High-frequency permittivity background (tuned as needed)
eps_inf = 1.82;                     % dimensionless
% ----------------- Wavelength grid -----------------
lambda_nm = (200:1:800)';           % nm
lambda_m  = lambda_nm * 1e-9;       % m
omega     = 2*pi*c ./ lambda_m;     % rad/s
% ----------------- Permittivity: single Lorentz oscillator ----------------
-
eps_array = eps_inf + omega_p2 ./ (omega0^2 - omega.^2 - 1i*gamma.*omega);
% ----------------- Refractive index -----------------
n_array = sqrt(eps_array);
% Enforce passive sign convention (n'' >= 0 for e^{-i wt})
flip_idx = imag(n_array) < 0;
```

```
n_array(flip_idx) = -n_array(flip_idx);
n_real = real(n_array);
n_imag = imag(n_array);  % corresponds to n'' under e^{-i wt}
% ---------------- Plot ----------------
figure('Color','w');
yyaxis left
plot(lambda_nm, n_real, '-', 'LineWidth', 1.5);
ylabel('n'' (real part)');
yyaxis right
plot(lambda_nm, n_imag, '-', 'LineWidth', 1.5);
ylabel('n'''' (imaginary part)');
xlabel('Wavelength (nm)');
title('Lorentz-oscillator simulation of complex refractive index');
grid on;
legend({'n''','n'''''}, 'Location','best');
```

Supplementary References

(1) Miller, D. A.; Xu, Y.; Highland, R.; Nguyen, V. T.; Brown, W. J.; Hong, G.; Yao, J.; Wax, A. Enhanced Penetration Depth in Optical Coherence Tomography and Photoacoustic Microscopy in Vivo Enabled by Absorbing Dye Molecules. *Optica* **2025**, *12* (1), 24–30.
(2) Zuo, T.; Tao, C.; Liu, X. Absorbing Molecules as Optical Clearing Agents Improve the Resolution and Sensitivity of Photoacoustic Microscopy. *Opt. Lett.* **2025**, *50* (7), 2282–2285.
(3) Jia, C.; Zhang, Z.; Shen, Y.; Hou, W.; Zhao, J.; Luo, J.; Chen, H.; Qi, D.; Yao, Y.; Deng, L.; Ma, H.; Sun, Z.; Zhang, S. Tartrazine-Enabled Optical Clearing for in Vivo Optical Resolution Photoacoustic Microscopy. *Biomed. Opt. Express* **2025**, *16* (6), 2504–2515.
(4) Narawane, A.; Trout, R.; Viehland, C.; Kuo, A. N.; Vajzovic, L.; Dhalla, A.-H.; Toth, C. A. Optical Clearing with Tartrazine Enables Deep Transscleral Imaging with Optical Coherence Tomography. *J. Biomed. Opt.* **2024**, *29* (12), 120501.
(5) Xu, M.; Yang, B.; Song, S.; Xu, T.; Yao, J.; Liu, Y.; Cui, Y.; Zhang, Y. Multi-Wavelength Photoacoustic Microscopy Enhanced by the High-Sensitivity Probe and Reversible Tissue Transparent Molecules. *Photonics Res.* **2025**, *13* (10), 2757.
(6) Liang, Y.; Meng, X.; Wang, C.; Ma, J.; Zhang, X.; Fan, F.; Zhu, J. Optical Coherence Tomography and Angiography Image Enhancement Using Optical Clearing Agent Tartrazine. *J. Biophotonics* **2026**, *19* (1), e202500297.
(7) Surkov, Y.; Timoshina, P.; Uvakin, I.; Shushunova, N.; Konovalov, A.; Kozlov, I.; Piavchenko, G.; Telyshev, D.; Meglinski, I.; Kuznetsov, S.; Tuchin, V. Computer-Guided Optical Clearing for Transcranial Laser Speckle Imaging of Cortical Blood Flow through Synergistic Tartrazine-Induced Cranial Bone Transparency. *J. Innov. Opt. Health Sci.* **2025**, No. 2540002. https://doi.org/10.1142/s1793545825400024.
(8) Lee, M. K. H.; Mizushima, K.; Zheng, P.; Tanwar, S.; Gupta, A.; Fujita, K.; Barman, I. Spectrally Silent and Optically Transparent: Clear-SiR for Deep Raman Biomolecular Sensing. *ACS Sens.* **2025**, *10* (10), 7702–7711.
(9) Yuan, N.; Ragab, S.; Chavez, L.; Pandey, V.; Intes, X. Evaluating Tartrazine as an Optical Clearing Agent for Fluorescence Lifetime Imaging. *Opt. Lett.* **2025**, *50* (24), 7588–7591.
(10) Toledano, P. M.; Zimmerman, G.; Tischler, Y. R. Concentration and Thickness Dependent Optical Transparency in Biological Tissues via Refractive-Index Modulation. *Opt. Express* **2026**, *34* (3), 5158–5168.
(11) Sun, T.; Su, J.; Zhao, Y.; Tie, X. Enhancing the Efficiency of Achieving Optical Transparency in Live Animals Using Absorbing Molecules. *J. Biomed. Opt.* **2026**, *31* (5), 054702.
(12) Minopoli, A.; Evangelista, D.; Marras, M.; Perini, G.; Augello, A.; Palmieri, V.; De Spirito, M.; Papi, M. Enhancing Photothermal Therapy Effectiveness via Tartrazine-Induced Optical Clearing of Biological Tissues. *Sci. Rep.* **2026**, *16* (1), 7553.
(13) Kong, J.; Wang, L.; Pi, S. Tartrazine-Enhanced Visible-Light OCT for Deep-Tissue Imaging. *Biomed. Opt. Express* **2026**, *17* (3), 1255.
(14) Wang, S.; Bao, J.; Chen, J.; Santacruz, S. R.; Estrada, J. B.; Fan, D. E.; Yang, J. Experimental Characterization of High-Strain-Rate Viscoelastic and Damage Behavior in Anisotropic Soft Materials Using Laser-Induced Inertial Cavitation. *Res. Sq.*, 2026. https://doi.org/10.21203/rs.3.rs-8840917/v1.
(15) Tie, X.; Sun, T.; Xiao, G.; Zhao, Y.; Su, J.; Xie, X.; Yin, W. Absorbing Molecules Make Both Abdomen and Back Transparent in Live Mice. *bioRxiv*, 2024. https://doi.org/10.1101/2024.10.28.620537.
(16) Dhodapkar, R. M. Isotonic Tartrazine Is Sufficient to Improve Transscleral Penetration of Optical Coherence Tomography. *bioRxiv*, 2025. https://doi.org/10.1101/2025.11.17.688647.
(17) Seong, D.; Yun, S.; Han, S.; Biswas, S.; Kim, B.; Remlova, E.; Razansky, D.; Kim, J.; Ou, Z.; Jeon, M. Beyond the Skin Barrier: Optical Clearing Enables Non-Invasive Cortex-Wide Optical Coherence Angiography in Mice in-Vivo. *bioRxiv*, 2026.

https://doi.org/10.64898/2026.03.02.709062.
(18) Paul, M. B.; Niknam, K.; Das, M. Enhancing Imaging Depth and Sensitivity in Reflectance Mode near Infrared Optical Imaging with Scatter Reducing Agents. *arXiv [physics.optics]*, 2026. https://doi.org/10.48550/arXiv.2602.00368.
(19) Krysa, M.; Chwastowicz, A.; Lenarcik, M.; Matrybak, P.; Zdankowski, P.; Trusiak, M. The Mixture of Glycerin with Tartrazine: A Solution to Reversibly Increase Tissue Transparency for in Vitro Quantitative Phase Imaging. *arXiv [physics.optics]*, 2026. https://doi.org/10.48550/arXiv.2602.09732.
(20) Shabbir, M. W.; Biswas, S.; Kajla, R.; Nadella, S.; Ou, Z. Designing Refractive Index Fluids of Food Dye for Light Propagation through Scattering Media. *MRS Commun.* **2025**. https://doi.org/10.1557/s43579-025-00729-6.
(21) Shabbir, M. W.; Asante-Asare, D.; Phillips, M.; Ou, Z. Transient Optical Clearing Using Absorbing Molecules for Ex Vivo and in Vivo Imaging. *J. Vis. Exp.* **2025**, No. 221. https://doi.org/10.3791/68629.
(22) Ou, Z.; Duh, Y.-S.; Rommelfanger, N. J.; Keck, C. H. C.; Jiang, S.; Brinson, K., Jr; Zhao, S.; Schmidt, E. L.; Wu, X.; Yang, F.; Cai, B.; Cui, H.; Qi, W.; Wu, S.; Tantry, A.; Roth, R.; Ding, J.; Chen, X.; Kaltschmidt, J. A.; Brongersma, M. L.; Hong, G. Achieving Optical Transparency in Live Animals with Absorbing Molecules. *Science* **2024**, *385* (6713), eadm6869.
(23) Keck, C. H. C.; Schmidt, E. L.; Zhao, S.; Liu, Z.; Zhang, L.-Y.; Cui, M.; Chen, X.; Wang, C.; Cui, H.; Brongersma, M. L.; Hong, G. Achieving Transient and Reversible Optical Transparency in Live Mice with Tartrazine. *Nat. Protoc.* **2025**. https://doi.org/10.1038/s41596-025-01187-z.
(24) Lu, K.; Xu, Y.; Miller, D. A.; Wang, W.; Gupta, D.; Yao, J.; Wax, A. Indocyanine Green (ICG) Enhances Penetration of 1300 Nm Optical Coherence Tomography Imaging for in Vivo Murine Skin. *Opt. Lett.* **2025**, *50* (17), 5226–5229.
(25) Trout, R. M.; Narawane, A.; Viehland, C.; Vajzovic, L.; Dhalla, A.-H.; Kuo, A. N.; Toth, C. A. Optical Clearing with Fluorescein for Transscleral Optical Coherence Tomography Imaging in Model Eyes. *Invest. Ophthalmol. Vis. Sci.* **2025**, *66* (8), 5501–5501.
(26) Trout, R. M.; Narawane, A.; Viehland, C.; Ownagh, V.; Draelos, M.; Dhalla, A.-H.; Kuo, A.; Toth, C. Optical Coherence Tomography with Fluorescein Optical Clearing for Transscleral Image Guidance. *bioRxivorg*, 2025. https://doi.org/10.1101/2025.07.01.661162.
(27) Keck, C. H. C.; Schmidt, E. L.; Roth, R. H.; Floyd, B. M.; Tsai, A. P.; Garcia, H. B.; Cui, M.; Chen, X.; Wang, C.; Park, A.; Zhao, S.; Liao, P. A.; Casey, K. M.; Reineking, W.; Cai, S.; Zhang, L.-Y.; Yang, Q.; Yuan, L.; Baghdasaryan, A.; Lopez, E. R.; Cooper, L.; Cui, H.; Esquivel, D.; Brinson, K.; Chen, X.; Wyss-Coray, T.; Coleman, T. P.; Brongersma, M. L.; Bertozzi, C. R.; Wang, G. X.; Ding, J. B.; Hong, G. Color-Neutral and Reversible Tissue Transparency Enables Longitudinal Deep-Tissue Imaging in Live Mice. *Proc. Natl. Acad. Sci. U. S. A.* **2025**, *122* (35), e2504264122.
